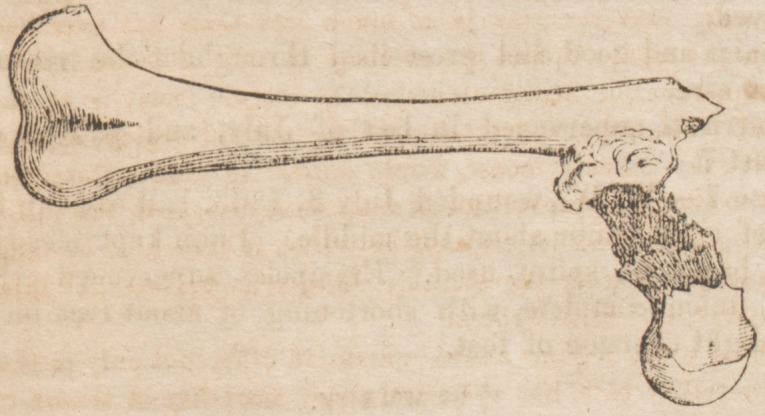# Confederate States Hospital Reports

**Published:** 1864-01

**Authors:** 


### CONFEDERATE STATES HOSPITAL REPORTS.

I.—*Statistics of Winder Hospital, Richmond, Va., from its organization, April* 1, 1862, *to December* 1, 1863. Surg. A. G. LANE, in charge.

| | | | |
|---|---|---|---|
| Total | number | admitted, | 39,997 |
| " | " | transferred, | 7,830 |
| " | " | returned to duty, | 20,720 |
| " | " | furloughed, | 6,675 |
| " | " | discharged, | 284 |
| " | " | deserted, | 1,138 |
| " | " | died, | 2,149 |

Ratio of mortality, $5\frac{27}{100}$ per cent.

---

II.—*Statistics of Chimborazo Hospital from Nov.* 1, 1861, *to Nov.* 1, 1863. Surg. J. B. McCAW, in charge.

| | | | |
|---|---|---|---|
| Total | number | admitted, | 47,176 |
| " | " | transferred, | 17,384 |
| " | " | returned to duty, | 17,845 |
| " | " | furloughed, | 4,378 |
| " | " | discharged, | 635 |
| " | " | deserted, | 846 |
| " | " | died, | 3,031 |

Aggregate ratio of mortality, $6\frac{62}{100}$ per cent.

Total number VULNUS SCLOPETICUM, 6,740—Deaths, 377.
Ratio of mortality, $5\frac{74}{100}$ per cent.

Total number FEBRIS TYPHOID,.......3,474—Deaths, 951.
Ratio of mortality, $27\frac{55}{100}$ per cent.

Total number PNEUMONIA,...........2,739—Deaths, 637.
Ratio of mortality, $20\frac{39}{100}$ per cent.

Total number ALL OTHER DISEASES, 34,223—Deaths, 1,965.

Ratio of mortality, $3\frac{1}{100}$ per cent.

III.—*Statistics of Compound Fracture of Femur. A report of cases of Compound Comminuted Fracture of the Thigh from Gun-Shot Wounds treated in Winder Hospital, Richmond, Va., since its organization, April 1, 1862, to December 1, 1863.* By Surg. A. G. Lane, in charge.

| No. | Nature and Seat of Wound. | Character of Operation, Time and Point of Same. | Treatment. | Result. |
|---|---|---|---|---|
| 1 | Fracture at middle third, | Amputation, flap operation, primary, at upper third, | ........................ | Died. |
| 2 | Fracture at lower third, | Amputation, circular operation, secondary, at middle third, | ........................ | Died. |
| 3 | Fracture at lower third, | Amputation, flap operaton, primary, at middle third, | ........................ | Recovered. |
| 4 | Fracture at lower third, | Amputation, circular operation, secondary, at middle third, | ........................ | Recovered. |
| 5 | Fracture at lower third, | Amputation, circular operation, secondary, at middle third, | ........................ | Recovered. |
| 6 | Fracture at upper third, | ........................ | Treated with double inclined plane, | Died. |
| 7 | Fracture at upper third, | ........................ | Treated with double inclined plane, | Died. |
| 8 | Fracture at upper third, | ........................ | Treated with double inclined plane, | Died. |
| 9 | Fracture at upper third, | ........................ | Treated with Smith's anterior splint, | Died. |
| 10 | Fracture at upper third, | ........................ | Treated with Smith's anterior splint, | Died. |
| 11 | Fracture at lower third, | ........................ | Treated with Smith's anterior splint, | Died. |
| 12 | Fracture at upper third, | ........................ | Treated with Smith's anterior splint, | Died. |
| 13 | Fracture at middle third, | ........................ | Treated with Smith's anterior splint, | Recovered. |
| 14 | Fracture at upper third, | ........................ | Treated with Smith's anterior splint, | Recovered. |
| 15 | Fracture at middle third, | ........................ | Treated with Smith's anterior splint, | Recovered. |
| 16 | Fracture at middle third, | ........................ | Treated with Smith's Anterior splint. | Recovered. |
| 17 | Fracture at middle third, | ........................ | Treated with Smith's posterior splint, | Died. |
| 18 | Fracture at lower third, | ........................ | Treated with long, straight splint, | Died. |
| 19 | Fracture at middle third | ........................ | Supported on pillows, | Died. |
| 20 | Fracture at upper third, | ........................ | Supported on pillows, | Died. |

SUMMARY.

| | Recovered. | Died. | Total. |
|---|---|---|---|
| Operations, primary, flap........................ | 1 | 1 | 2 |
| Operations, secondary, circular.................. | 2 | 1 | 3 |
| Amputation at upper third......................... | 0 | 1 | 1 |
| Amputation at middle third........................ | 3 | 1 | 4 |
| Number treated with double inclined plane... | 0 | 3 | 3 |
| Number treated with Smith's anterior splint.. | 4 | 4 | 8 |
| Number treated with Smith's posterior splint.. | 0 | 1 | 1 |
| Number treated with long, straight splint..... | 0 | 1 | 1 |
| Number supported on pillows...................... | 0 | 2 | 2 |
| Number of fractures at upper third............. | 1 | 7 | 8 |
| Number of fractures at middle third........... | 2 | 3 | 5 |
| Number of fractures at lower third............. | 0 | 2 | 2 |

Number recovered, 7; number died, 13; total number treated, 20.

IV.—*Report of cases of Gun-shot Fracture of Femur treated without operative procedure, Chimborazo Hospital.*

No. 4.—Surgeon Wm. A. Davis, in charge.

Case 1.—J. C., wounded June 29th, 1862, ball entering anterior aspect of left thigh, about two inches above upper edge of patella, and escaping on posterior aspect a little higher up, fracturing femur in its lower third. Limb placed in long fracture box, without extension. Patient did well, and was furloughed August 31, at which time the bone was firmly united, with very slight shortening.

Case 2.—S. H., wounded June 27, 1862, by three separate balls, fracturing right femur in its upper, middle and lower thirds, the bone being crushed throughout most of its extent. He had also flesh wounds of left leg and left shoulder.

Fractured limb made as comfortable as possible, and patient treated with tonics and nutritious diet. Died July 29th.

Case 3.—M. M., wounded June 27th, 1862, ball entering upper part of lower third of right thigh, causing oblique fracture at this point, and separating a section of the bone three-quarters of an inch in length, which was removed.

He had also a gun-shot fracture of upper third of left radius, and a flesh wound of left side exposing two false ribs.

He could bear no extension; was made as comfortable as possible, well fed, and treated with tonics and opiates. Recovered with shortening of three or four inches, and was furloughed in January, 1863.

Case 4.—J. B., wounded July 1, 1862, ball shattering the right femur at junction of middle and upper third, and remaining in the thigh. Patient died August 5th.

Case 5.—J. C., wounded July 3, 1863, ball entering on outer side of left thigh, about three inches below trochanter major, causing fracture of femur in lower part of upper third. Nov. 10, union complete, with shortening of two inches.

Case 6.—W. T., wounded July 1, 1862, ball entering middle of left thigh, passing transversely through it, causing fracture of femur at this place. Smith's anterior splint was applied and continued for fifteen days, when abscess formed in the posterior aspect of the the thigh, and the splint was removed.

Tonics and good diet prescribed throughout the treatment of the case.

Diarrhœa supervened in last of July, and patient died August 7.

Case 7.—W. W., wounded July 3, 1863, ball causing fracture of right femur about the middle. Limb kept steady by sand bags; no splint used. Erysipelas supervened. Nov. 18th, union complete, with shortening of about two inches, and slight eversion of foot.

SUMMARY.

Case 1. Wounded June 29, 1862; fracture of lower third; recoevred; shortening three-quarters of an inch.

Case 2. Wounded June 27th, 1862; fracture of upper, middle and lower thirds: died July 29th; complicated with flesh wounds of left leg and left shoulder.

Case 3. Wounded June 27th, 1862; fracture of lower third; recovered; shortening three to four inches; complicated with gun-shot fracture of radius and wound of side.

Case 4. Wounded July 1st, 1862; fracture of middle and upper thirds; died August 5th.

Case 5. Wounded July 3d, 1863; fracture of upper third; recovered; shortening two inches.

Case 6. Wounded July 1st, 1862; fracture of middle third; died August 7th; complicated with diarrhœa.

Case 7. Wounded July 3d, 1863; fracture of middle third; recovered; shortening two inches; complicated with erysipelas.

| | Recovered. | Died. | Total. |
|---|---|---|---|
| Fracture of upper third................ | 1 | 2 | 3 |
| Fracture of middle third........... ... | 1 | 1 | 2 |
| Fracture of lower third................ | 2 | 0 | 2 |

No. 2.—Surgeon S. E. HABERSHAM, in charge.

Case 1.—Gun-shot wound of right thigh, with compound comminuted fracture of femur three inches below trochanter major—bone uniting with great deformity and shortening. Death from arterial hemorrhage 328 days after receipt of wound.

J. B., a private, company "F," 19th Georgia regiment infantry, æt. 23, was admitted into this hospital on the 16th Dec., 1862, with compound comminuted fracture of right thigh from a Minie ball; also a gun-shot wound on anterior aspect of same limb, grazing tibia about six inches above ankle, and a severe contused and lacerated wound from fragment of shell on left gluteal region. In consequence of these wounds and their serious character, it was found impossible to treat the fracture with splints or extensions, and the patient was placed in as comfortable a position as possible, supported by pillows and pads. Various means of treatment were suggested by different surgeons, but all plans calculated to resist muscular contraction caused the patient so much pain and inconvenience that their use was eventually abandoned, and he was treated by position and pillows alone. The wound continued to suppurate freely from both orifices until the middle of July last, which time they healed, and patient was enabled to walk about the yard with the assistance of a crutch. In the hope of enabling him to exercise himself with less inconvenience, a starch bandage was applied to the limb, which remained on several weeks, but he found it irksome, and it was removed at his request The wound shortly after opened again, and irritative fever occurred, from which he became much emaciated and debilitated.

On the 3d November, a profuse hemorrhage occurred at night, which was arrested by pressure upon the os pubis, but the patient gradually sank from the loss of blood, and died on the night of the 5th.

A post mortem examination revealed the following facts: Bone fractured three inches below trochanter major, which united, as per diagram, several small spiculæ of bone lying in proximity to artery, one of which had punctured the femoral artery just below its bifurcation.

A consultation of surgeons was called, who decided that ligation of artery was inexpedient because of the certainty of gangrene of limb resulting therefrom.

Case 2.—J. M., company "D," 31st Georgia, æt. 18, gun-shot wound of lower-third of thigh, compound comminuted fracture.

J. M., company "D," 31st Georgia regiment infantry, æt. 18, was wounded June 27th, 1862, and was admitted into this hospital on July 1st, with compound comminuted fracture of lower-third of thigh, ball entering and passing through antero posteriorly. At the time of admission there was much inflammation of limb. The limb was supported upon pillows, and cold water freely applied. In a few days he was transferred to private quarters, when Smith's anterior splint was applied. Was sent home on 15th November in charge of his family physician, the wound at the time discharging moderately; partial union was supposed to have taken place. When last heard from this patient was recovering.

Case 3.—N. T. T., company "F," 1st South Carolina regiment infantry, wounded June 27th, 1862.

N. T. T., a private, company "F," 1st South Carolina regiment infantry, entered hospital June 30th, with compound comminuted fracture of upper-third of femur. The whole limb was œdematous, and thigh extensively infiltrated. Cold water dressing was used, but the patient died July 7th of pyæmia.

Case 4.—J. D. L., wounded June 30th, 1862.

J. D. L., a private of company "F," 5th regiment South Carolina infantry, aged 22 years, was admitted July 2d, with compound comminuted fracture of upper middle-third of thigh. He was treated with cold water dressing, limb resting on pillows for several days. Smith's anterior splint was finally applied, and the patient furloughed nearly recovered.

Case 5.—W. L. C., wounded June 30th, 1862.

W. L. C., a private of company "E," 11th Alabama regiment infantry, was admitted July 2d, with compound fracture of middle-third of thigh Was treated with cold water dressing until œdema and infiltration subsided, and Smith's anterior splint was then applied, and cold dressing continued. Patient recovered with an inch and a half of shortening of limb and thigh slightly bent antero posteriorly.

Case 6.—W. O. B., wounded July 1st, 1863.

W. O. B., company "N," 12th regiment Virginia infantry, was admitted July 3d, with compound fracture of upper-third of thigh; œdema of limb very extensive. Cold water was used while in No. 2 Division, but he was transferred to No. 1 July 9th, and died in that hospital from the effects of the wound.

Case 7.—J. A., wounded 27th June, 1862.

J. A., a private of company "B," 38th regiment Virginia infantry, was admitted July 3d, 1862, with compound comminuted fracture of upper-third of thigh bone completely crushed; great prostration. Patient died of exhaustion July 6th, 1862.

Case 8.—Compound fracture of left thigh in middle-third from gun-shot wound, and recovery of same with very little shortening or deformity.

Private J. A. G., company "K," 35th regiment Georgia infantry, was wounded on the 13th December, 1862, and admitted into the hospital on the 15th inst., with compound fracture of middle-third of left thigh from a Minie ball, entering diagonally and passing horizontally through the limb. Was treated with cold and warm water dressing, without extension, the limb simply supported on pillows, with short splint applied. This patient recovered without a bad symptom, and was transferred to Columbus, Ga., on the 16th April, 1863. Three-quarters of an inch shortening.

SUMMARY.

| | |
|---|---|
| Upper-third, cases,........5 | —Deaths,..................5 |
| Middle-third, cases,.......2 | —Recovered,..............2 |
| Lower-third, cases,........1 | —Recovered,..............1 |

No. 5.—Surgeon E. M. SEABROOK, in charge.

Case 1st, No. 2296.—Private S. W. Brown, company "A," 2d Florida; wounded May 31st, 1863; admitted June 1st; compound fracture of thigh in lower-third; general health bad; aged 45 years. In this case there was shortening of the limb about an inch and a half. He left the hospital on furlough, August 21st, 1862.

Case 2d, No. 2305.—Thomas Mullen, Sergeant-Major of

11th Mississippi; wounded June 1st, 1862; admitted June 1st, 1862; amputation performed the same day; compound fracture of thigh immediately above the condyles of the femur. This patient was about 26 years old; stout and healthy. Discharged from service July 7th, 1862.

Case 3d, No. 2315.—A. Trexler Cross, Lee's battery, N. C. artillery; wounded May 31st, 1862; admitted June 1st; compound fracture of thigh immediately below the trochanters. The general health of this patient was remarkably good; aged 24 years; furloughed July 6th, 1862.

Case 4th, No. 2696.—Henry Carter, private company "A," 6th Virginia infantry; wounded June 21st, 1862; received in hospital June 22d; fracture of femur immediately above the knee; amputation performed June 23d, at the junction of the middle and lower-thirds; general health bad; aged 20 years. Necrosis of the stump occurred in this case, but the result was favorable, the stump proving a good one; transferred to Palmyra hospital, August 15th, 1863.

Case 5th, No. 2876.—B. H. Heaslitt, private company "K," 10th Alabama; wounded June 29th, 1862; admitted June 30th; compound fracture of the middle of thigh; general health good; aged 18 years. In this case there was shortening of the limb about two inches. Furloughed Aug. 21st, 1862.

Case 6th, No. 2922.—Eli Helms, private company "B," 23d North Carolina; wounded June 29th, 1862; admitted June 30th; compound fracture of thigh just below the middle; general health bad; aged 30 years; discharged July 24th, 1862.

Case 7th, No. 2996.—J. W. Suttles, private company "K," 8th Alabama; wounded June 30th, 1862; admitted July 2d; fracture of femur at its middle; aged 29 years; died July 3d. This man appeared to have died from constitutional shock.

Case 8th, No. 3020.—John Pate, private company "F," 18th North Carolina; wounded June 30th, 1862; admitted July 2d; compound fracture of thigh in upper-third; general health not good; aged 30 years; died July 26th, 1862.

Case 9th, No. 3008.—A. Wright, Lieut. company "A," 2d Florida; wounded July 1st, 1862; admitted July 2d; compound fracture of upper-third of thigh; general health not good; aged 23 years; died July 13th, 1862.

In all the above cases pillows were adjusted comfortably to the injured limbs.

SUMMARY:

Upper-third—cases, 3; deaths 2; recovered, 1.
Middle-third—cases, 2; recovered, 2.
Lower-third—cases, 4; recovered, 4.

N. B—Two of the four cases in lower-third operated on; two not operated on.

## Figures and Tables

**Figure f1:**